# Role of the bHLH transcription factor TCF21 in development and tumorigenesis

**DOI:** 10.1590/1414-431X202010637

**Published:** 2021-03-15

**Authors:** C.F.P. Lotfi, B.S. Passaia, J.L. Kremer

**Affiliations:** 1 Instituto de Ciências Biomédicas, Departamento de Anatomia, Universidade de São Paulo, São Paulo, SP, Brasil

**Keywords:** TCF21, Cancer suppressor genes, Growth and development, Cancer, Tumorigenesis, Neoplasm metastases

## Abstract

Transcription factors control, coordinate, and separate the functions of distinct network modules spatially and temporally. In this review, we focus on the transcription factor 21 (TCF21) network, a highly conserved basic-helix-loop-helix (bHLH) protein that functions to integrate signals and modulate gene expression. We summarize the molecular and biological properties of TCF21 control with an emphasis on molecular and functional TCF21 interactions. We suggest that these interactions serve to modulate the development of different organs at the transcriptional level to maintain growth homeostasis and to influence cell fate. Importantly, *TCF21* expression is epigenetically inactivated in different types of human cancers. The epigenetic modification or activation and/or loss of *TCF21* expression results in an imbalance in TCF21 signaling, which may lead to tumor initiation and, most likely, to progression and tumor metastasis. This review focuses on research on the roles of TCF21 in development and tumorigenesis systematically considering the physiological and pathological function of TCF21. In addition, we focus on the main molecular bases of its different roles whose importance should be clarified in future research. For this review, PubMed databases and keywords such as TCF21, POD-1, capsulin, tumors, carcinomas, tumorigenesis, development, and mechanism of action were utilized. Articles were selected within a historical context as were a number of citations from journals with relevant impact.

## Introduction

### Definition and classification of basic helix-loop-helix (bHLH) proteins

The *bHLH* genes are conserved transcription factors that have been reported in eukaryotic organisms and unicellular organisms. When bHLH proteins are expressed in determined cell types, they induce a number of genes and specific phenotypes. A bHLH domain is composed of two regions, a basic region for binding to a target DNA sequence and a helix‐loop‐helix (HLH) motif, a structure comprising two α‐helices separated by a loop of variable length. bHLH motifs mediate dimerization, producing homo- or heterodimers (1,2). bHLHs have been categorized into different classes based on molecular phylogenetic relationships, expression patterns, dimerization selectivity, DNA-binding specificities, and protein structures (3,4). The classification that divides bHLH proteins into seven classes based on the presence of additional domains, the expression pattern, and the observed transcriptional function has been commonly employed (1,5). Class I HLH proteins are abundantly expressed and bind DNA as either homodimers or heterodimers. These HLH proteins were named E proteins (E12, E47, E2-2, HEB, TCF4), since they bind to Ephrussi-box (E-box) sequences (CANNTG) (6). Class II HLH proteins (MyoD, myogenin, NeuroD1-D2, MYF5-6) bind DNA as either homodimers or heterodimers with E proteins. In contrast to Class I HLH protein, which is expressed in many tissues, Class II protein expression is tissue-specific. Both Class I and Class II of the bHLH transcription factors (TFs) do not possess additional domains. Class III proteins (c-Myc) also contain a leucine zipper dimerization domain and function as either transcriptional activators or repressors, which regulate oncogenic transformation, apoptosis, and cellular differentiation. Class IV HLH proteins (Mad, Max, and Mxi) form heterodimers with c-Myc and regulate its activity but are also able to create homo- and heterodimers with each other. These proteins bind to CACGTG or CATGTTG E-box sites, but Mad/Max dimers are transcription regulators lacking a transactivation domain (TAD) that act on transcription otherwise. In Class V, HLH proteins (Id proteins) lack a basic region and antagonize the DNA activity of their bHLH partners. Class VI HLH proteins (Hes protein) present proline residues in their basic regions. Class VI HLH proteins recognize a unique sequence, CACGCG or CACGAG, and predominantly achieve transcriptional repression by binding to Groucho. Finally, the class VII HLH proteins (including members of circadian clock proteins and hypoxia) show PAS domains and bind predominantly to ACGTG or GCGTG sequences and function as transcriptional repressors (5,7).

### TCF21 identification

In 1998, a novel member of the bHLH transcription factor family was identified as essential during murine embryonic development of mesodermal tissues. Firstly, it was known as epicardin (8), capsulin (9,10), or POD-1 (11), and currently as transcription factor 21 (TCF21). TCF21 is a bHLH class II transcription factor expressed in brachial muscle precursors and mesenchymal cells at sites of epithelial-mesenchymal interactions in the kidney, lung, intestine, pancreas, and spleen and in the developing respiratory, gastrointestinal, urogenital, and cardiovascular systems during embryogenic development. The expression levels of Tcf21 rapidly decrease in postnatal tissues, except in interstitial cells of kidney, lung, and heart. Knockout Pod1 mice die at birth due to lung, kidney, and cardiac defects and display gonadal, gastric, and splenic dysgenesis, suggesting a crucial role in embryogenesis (12–16). *TCF21* is encoded by a gene located on human chromosome 6q23-q24 and can bind to the consensus E-box sequence to form a heterodimer with E12, also known as HEB or TCF12 (17,18). However, there are other putative interactants that have not been fully validated to date as shown in Table 1 (19–23).

**Table 1:** Possible interactions of transcription factor 21 (TCF21).

Interactant	Full name	Experiment type	Experimental model	References
TCF12; *HEB; CRS3	Transcription factor 12	Affinity Capture Western; Yeast 2 Hybrid	HEK293 cells	18
APLP1	Amyloid-like protein 1	Yeast 2 Hybrid	COS-1 and HEK293 cells	19
LMO4	LIM domain only 4	Yeast 2 Hybrid	HepG2 cells	20
TCF3, E2A, E47, ITF1, VDIR, bHLHb21	Transcription factor 3	*In vivo*, *In vitro*, Yeast 2 Hybrid	Mouse, HEK293 cells	18–21
APEX1	Apurinic/apyrimidinic endodeoxyribonuclease1 APEX nuclease	Yeast 2 Hybrid	CHO-K1 cells	22
GTF3C5	General transcription factor IIIC subunit 5	Yeast 2 Hybrid	CHO-K1 cells	22
HOXB6	Homeobox B6	Yeast 2 Hybrid	CHO-K1 cells	22
KLF15	Kruppel-like factor 15p	Yeast 2 Hybrid	HEK293 cells	23
TCF4	E2-2; transcription factor 4	Affinity Capture Western; Yeast 2 Hybrid	HEK293 cells	18

*HEB: transcription factor 12; CRS3: craniosynostosis-3; E2A/E47: immunoglobulin enhancer-binding factors E12/E47; ITF1: immunoglobulin transcription factor 1; VDIR: VDR interacting repressor; bHLHb21: class B basic helix-loop-helix protein 21.

### TCF21 function

The functions and/or activities attributed to TCF21, inferred from sequence or structural similarity, sequence alignment, sequence model, direct assay, or physical interaction, are DNA-binding transcription activator, RNA polymerase II-specific, E-box binding, androgen receptor binding, bHLH transcription factor binding, histone deacetylase binding, and protein dimerization activity (24,25). Moreover, TCF21 is related to distinct and specific biological processes, such as development of the reproductive system (Sertoli cell differentiation and sex determination), urinary system (glomerulus development, metanephric mesenchymal cell differentiation, and glomerular capillary formation), respiratory system (lung morphogenesis and bronchiole and diaphragm development), spleen, adrenal gland, and digestive tract. In addition, TCF21 is involved in the negative and positive regulation of transcription by RNA polymerase and the negative regulation of the androgen receptor signaling pathway. In PubMed in early 2020, TCF21 was cited in 53 high-impact articles, and in 33 of them, a physiological or molecular function of TCF21 was cited.

### Regulation of TCF21 expression

**Figure 1:**
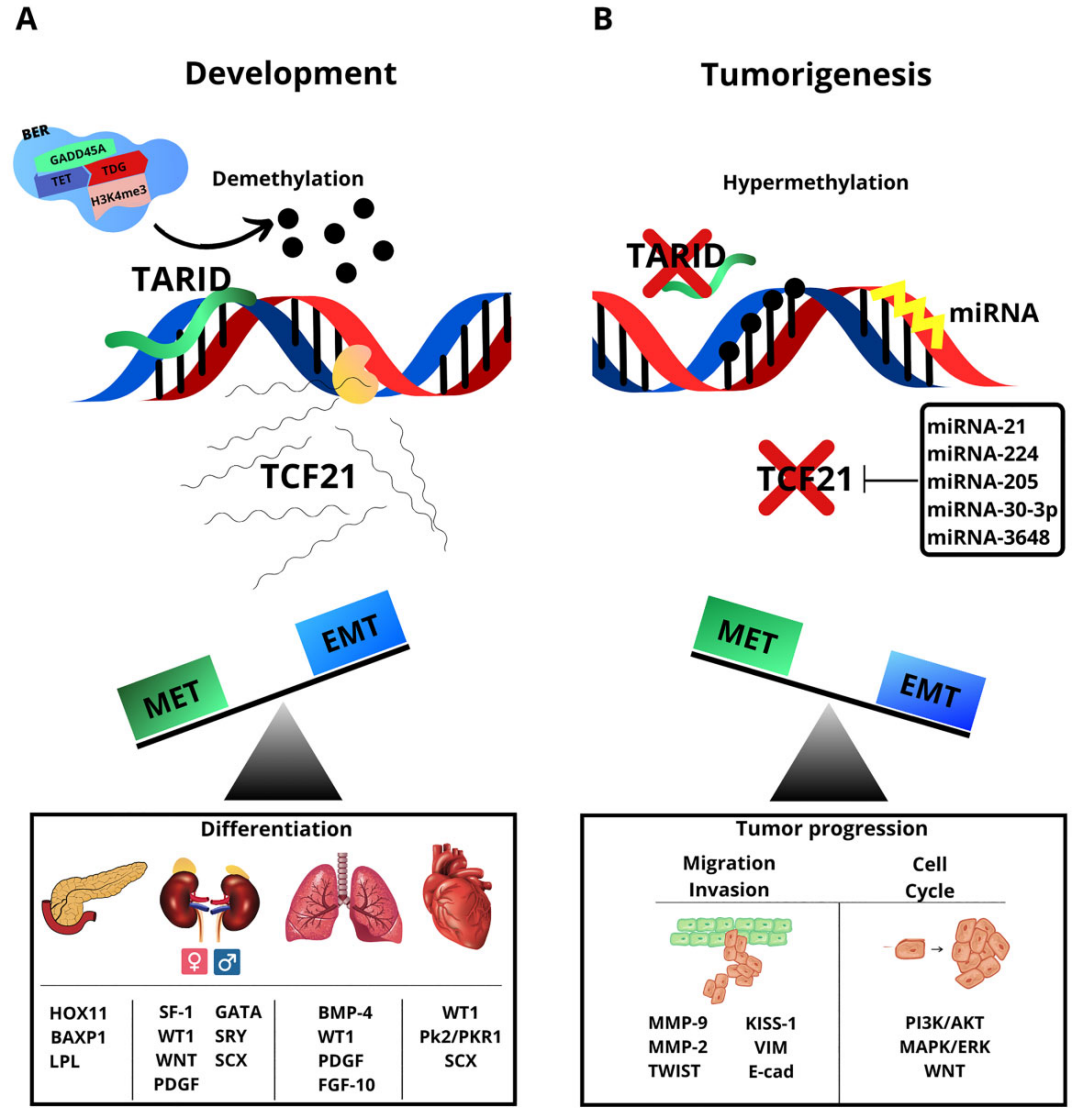
Transcription factor 21 (TCF21) participates in development and tumorigenesis through distinct mechanisms. **A**, In development, TARID recruits components of the BER demethylation pathway and activates TCF21 expression by inducing promoter demethylation. Expression of TCF21 contributes to mesenchymal-epithelial transition (MET) and embryogenic development/differentiation of organs through different factors. **B**, In tumorigenesis, TCF21 is hypermethylated, and silencing by microRNAs (miRNAs) may contribute to epithelial-mesenchymal transition (EMT) and tumor progression. TARID: TCF21 antisense RNA inducing promoter demethylation; BER: TDG dependent base excision repair; GADD45A: growth arrest and DNA damage inducible alpha; TET: ten eleven-translocation family of dioxygenases; TDG: thymine DNA glycosylase and trimethylation of histone H3 at lysine 4 (H3k4me3).

The expression of TCF21 seems to be regulated by epigenetic mechanisms, such as methylation, histone acetylation, and SUMOylation, as well as noncoding RNAs (ncRNAs), mostly described in tumor cells. The three exons encoding the human *TCF21* gene are associated with methylation of CpG islands. There is also evidence that the long non-coding RNA (lncRNA) *TCF21* antisense RNA-inducing demethylation, called TARID transcribed by exon 3, is associated with the CGI3 methylation island and is responsible for the demethylation of the CGI1 island in *TCF21* and activates *TCF21* expression by inducing promoter demethylation in melanomas (26) as described in Figure 1. TARID associates with the *TCF21* promoter, forming an R-loop, a structure that acts as an anchoring support for adapter protein GADD45A, which recruits essential components of the base excision repair (BER) pathway, TDG and TET, responsible for promoting DNA demethylation by oxidation and replacement of 5-methylcytosine with 5-cytosine (27,28). TARID also recruits histone acetyltransferase enzymes that add three methyl groups to lysine 4 of the histone 3 protein (H3K4me3), enabling the remodeling and opening of chromatin to access transcription factors (29). MicroRNAs (miRNAs), such as miRNA-21, miRNA-224, miRNA-205, miRNA-30-3p, and miR-3648, are also related to *TCF21* regulation (Figure 1) and are involved in the silencing of genes in human tumors (30–32). Inhibition of *TCF21* expression was also associated with an increase of risk of coronary artery disease in a Chinese population (33). More recently, a study suggested that CXC chemokine ligand 12 (CXCL12) activates the GSK3β/β-catenin/TCF21 signaling pathway through CXCR4, a specific receptor for CXCL12, inhibiting cholesterol efflux from macrophages and promoting atherosclerosis (34).

## TCF21 in development

First detected in visceral glomerular epithelial cells (podocytes) in the mouse kidney and with its expression related to the onset of podocyte differentiation, the observed bHLH transcript factor was named POD-1 (11). In the meantime, a bHLH transcription factor was called capsulin after its expression had been identified specifically in mesodermic-derived cells that encapsulate the heart and tubular structures in the lungs, kidneys, and intestine during mouse embryogenesis (9,10). In murine heart development, a bHLH transcription factor was found to be expressed in the epicardium and endocardium and was given the name epicardin, since it is involved in cardiac organogenesis (8). This section will discuss the important findings about the TCF21 transcription factor in the development of different tissues and organs illustrated in Figure 1.

## TCF21 in kidney development

TCF21 is persistently expressed from the early to the later stages of mouse kidney development in mesenchymal cells adjacent to the ureteric epithelium and in podocytes (11,35). The presence of TCF21 induces mesenchymal cells to condense followed by the mesenchymal-epithelial transition process. To determine the function of *Tcf21 in vivo,* a lacZ-expressing null Pod1 allele was generated. Null mutant mice are born but die with severely hypoplastic lungs and kidneys (12). The inhibition of *Tcf21* expression resulted in decreased condensation of mesenchymal cells, which are necessary for the formation of nephron tubules. Additionally, TCF21 seems to be important for podocyte migration and differentiation around glomerular capillaries (36). During glomerular maturation, the Wilms tumor suppressor gene (*WT1*) is expressed in the metanephrogenic mesenchyme, which positively regulates *Tcf21*, inducing podocyte differentiation (36,37). TCF21 may regulate differentiation through the inhibition of cell cycle arrest-promoting factors in the HEK293 cell line, such as p21, which promotes the expansion of undifferentiated precursor cells and Gdnf-Ret-Wnt1 pathway activation required for mouse branching morphogenesis (25,38).

## TCF21 in heart development

Capsulin/*Tcf21* was identified in different cell populations during murine heart development. Initially detected in pericardial mesoderm at approximately embryonic day (E) E8.5 dpc (days post-coitum), the cells expressing capsulin migrate to the heart surface and originate the visceral pericardium, smooth muscle and endothelial cells of coronary vessels (9,10). During mouse epicardial development, WT1, TCF21, TBX18, SEMA3D, and SCX mediate differentiation in a group of pro-epicardium cells (39,40), which provide multipotent progenitor cells for cardiac lineages, including pericytes, fibroblasts, and coronary smooth muscle cells (41,42). TCF21 seems to inhibit prockineticin-2/PKR1 signaling (Figure 1), promoting the differentiation of cardiac progenitor cells (39). Depletion of Tcf21 results in disruption of pro-epicardial cell specification and in failure of mature epithelial epicardium formation during early stages of epicardial development in Xenopus (43).

## TCF21 in lung development

Mice with the lacZ-expressing null *Pod1* allele present severely hypoplastic lungs and die at birth (12). During pulmonary embryogenesis, *Tcf21* is expressed in progenitor cells at the embryonic stage of E11.5 mesenchymal cells and regulates the expression of bone morphogenetic protein-4 (BMP-4), which is responsible for epithelial respiratory tract differentiation (12) (Figure 1). *Tcf21* is expressed in a subpopulation of fibroblasts and lipofibroblasts, and is co-expressed with fibroblast growth factor 10 (FGF10), a fibroblast differentiation marker, and platelet-derived growth factor α receptor (PDGFs) (44,45). TCF21 regulates human lipofibroblast activation and fetal lung maturation (46). TCF21 overexpression in primary neonatal pulmonary fibroblasts increases intracellular lipids responsible for activating the synthesis of surfactants and phospholipids in adult rat alveolar type II cells in culture (47).

## TCF21 in gonads and adrenal development

The murine adrenal and gonads have a common developmental origin, known as adrenogonadal primordium (AGP), detected at approximately E9.5 dpc (48). By E10.5 dpc, adrenal (AP) and gonadal primordium differentiate separately. During gonadal development, TCF21 is indispensable for ovarian and testicular differentiation and sexual differentiation. *Tcf21* knockout (KO) mice show gonadal failures, vascular abnormalities, and sex indistinction during embryogenesis, while mutant Tcf21 mice show gonadal dysgenesis (15,16). During mouse male sex determination, the testis-determining factor SRY promotes Sertoli cell differentiation and consequent gonadal development. *Tcf21* is a direct target of SRY (49), while the bHLH gene scleraxis (Scx) is a target of TCF21 (Figure 1). Therefore, the SRY-TCF21-SCX cascade participates in the initial molecular events of Sertoli cell differentiation and mouse testis development (50).

After differentiation of gonads, the expression of *Tcf21* increases in mouse males after E11.5 dpc and in females after birth. TCF21 regulates the expansion of progenitor gonadal cells by inhibiting the steroidogenic factor *Sf-1*, which is involved in the sexual differentiation of the gonads and the regulation of steroid hormones (15,16).

During mouse adrenal gland development, an AGP cell group that expresses higher levels of SF-1 migrates to the dorsomedial region to form AP, which settles ventrolateral to the aorta (51). Approximately 48 dpc and E13 in mice, neural crest cells migrate from the neural tube and invade the AP during development, differentiating into the catecholamine-producing chromaffin cells of the adrenal medulla (52). At approximately 52 dpc in humans and E14.5 in mice, the adrenal cortex becomes encapsulated with a fibrous layer of cells (53). While SF-1 is continuously expressed during adult life, *Sf-1* expression is driven by fetal adrenal-specific enhancer (FAdE) during AP development. FAdE becomes inactive when the adrenal gland differentiates, suggesting that distinct mechanisms are involved in *SF-1* expression in mouse fetal and adult adrenal glands (54).

Different studies have shown the presence of adrenocortical stem and/or progenitor/proliferative cells located in the outermost layers of mouse adrenal cortex in the subcapsular region. However, cell lineage tracing studies have shown definitive evidence of a centripetal change in adrenocortical cells between zones (55). Cellular and molecular studies provided evidence that the specific paracrine/autocrine signaling pathways activated are involved in adrenocortical homeostasis. The Wnt and hedgehog (HH) pathways are well-studied and characterized pathways activated to play a role in mouse adrenocortical homeostasis. Pathway activity is regulated by cortical and stromal cell crosstalk that maintains organ homeostasis. In addition to Wnt and HH pathways, other mesenchymal-like cell lineages have also been identified in the capsule and the stroma. In the adrenal capsule, cells expressing the Wilms tumor protein homolog (WT1), a transcriptional regulator of adrenal and gonad organogenesis, with AGP features were observed (37). Ectopic expression of WT1 prevents the differentiation locking of adrenocortical steroidogenic cells in progenitor status. In addition, *Tcf21* and *Gli1* are direct targets of WT1. Moreover, cell lineage tracing analyses identified a long-living progenitor population in the adrenal cortex expressing WT1, GATA4, GLI1, and TCF21 that can generate steroidogenic cells *in vivo* (Figure 1). These cells may also have important roles in adrenal gland maintenance. Wood et al. (56) identified cells expressing TCF21 in the mouse adrenal cortex at E9.5 until E14.5 when TCF21 expression is restricted to the adrenal capsule. Lineage-tracing experiments showed that before encapsulation, TCF21-expressing cells give rise to both non-steroidogenic capsular cells and steroidogenic cortical cells. The TCF21-expressing capsular cells give rise to a population of stromal cells in adult mice.

## TCF21 in the development of other tissues

Lu et al. (13 [Bibr B14]) reported that mice homozygous for the *Tcf21* null mutation fail to form a spleen. TCF21 seems to control morphogenetic expansion of splenic differentiation, and when it is absent, spleen precursor cells undergo apoptosis. Moreover, TCF21 controls an essential early step in spleen organogenesis among the *Hoxl1* and *Bapx1* genes, with both playing a critical role in murine spleen embryogenesis (57,58).

TCF21 may play an important role in regulating white adipose tissue development. Microarray analyses showed that *Tcf21* is expressed in murine white pre-adipocytes, but it is not expressed in brown pre-adipocytes (59). Subsequent reports have shown that *TCF21* mRNA decreases during the transition from white to brown human adipocytes (60). Moreover, TCF21 expression is significantly higher in the visceral adipose tissue of obese mice than of normal mice (61). Recently, a study using chicken lines showed a significant decrease in TCF21 levels in the abdominal fat of lean broilers compared with fat broilers. *In vitro*, *Tcf21* increased pre-adipocyte differentiation, whereas *Tcf21* knockdown and overexpression attenuated and promoted pre-adipocyte differentiation, respectively, which changed lipid droplet accumulation. Moreover, ChIP and luciferase analyses showed that TCF21 controls the transcription of lipoprotein lipase (LPL) by directly binding to the E-box motif in the *Lpl* promoter (62).

## TCF21 in the tumorigenic process

This part of the review focuses on advances in the understanding of the role of TCF21 in various tumors. TCF21 is involved in tumor initiation, invasion, metastasis, and apoptosis with the molecular mechanisms largely undefined (Figure 1). The pathological function and molecular basis of TCF21 have been considered, and studies have shown distinct biological roles and some directions for research. In 2006, Smith et al. (63) described for the first time that the promoter region on human chromosome 6q23-q24 of *TCF21* was hypermethylated in non-small-cell lung cancers (NSCLC) and head and neck squamous cell carcinomas. Since then, *TCF21* has been studied in other types of human cancers and is considered a tumor suppressor gene in melanomas, renal cancer, adrenocortical carcinomas, colorectal tumors, gastric cancer, and hepatocellular carcinomas (18,). In general, the proposition is that *TCF21* is downregulated primarily by DNA hypermethylation, rather than genetic mutations, in malignant tumors. DNA methylation can be reversed, and transcription can be restored, enabling TCF21 to be a therapeutic target. This section will discuss the important findings about the TCF21 transcription factor in tumorigenesis, what is unknown about its function, and what needs to be elucidated. These issues are illustrated in Figure 1.

## TCF21 in lung cancer

The application of genomic scanning to obtain DNA methylation profiling in the region of recurrent loss of heterozygosity at human chromosome 6q23-q24 identified the *TCF21* gene in lung cancer (63). In NSCLC, *TCF21* is aberrantly methylated. The prevalence of *TCF21* methylation in primary lung adenocarcinoma samples was 81%, higher than other methylated genes found in the 6q12-q27 locus (70). In addition, the ability of TCF21 to regulate mesenchymal-epithelial transition is lost in lung carcinomas. Moreover, exogenous expression of *TCF21* in cells that have silenced endogenous *TCF21* showed impaired tumorigenic properties *in vitro* and *in vivo* (63). In 2011, Richards et al. (71) concluded that *TCF21* hypermethylation and impaired *TCF21* expression are specific and frequent in NSCLC cells, even in early-stage disease, thereby making *TCF21* a potential biomarker for early-stage NSCLC. Demethylation of the *TCF21* promoter in H1299 cells (NSCLC cell line) by 5-aza-2'-deoxycytine (5-Aza) resulted in a higher level of *TCF21* expression, leading to a loss of cell viability and invasion ability and an increase in cell apoptosis. In addition, inhibition of autophagy by 3-methyladenine (3-MA) increased *TCF21* expression, suggesting that autophagy may also regulate *TCF21* expression (72).

Analysis of LINC00163 levels revealed that a novel lncRNA was significantly downregulated in metastatic lung cancer tissues compared with non-metastatic tissues. Functional studies showed that LINC00163 induces TCF21 expression by recruiting ARID1A to the *TCF21* promoter, suggesting a mechanism of lung cancer progression (73).

## TCF21 in genitourinary tumors

After gene expression assessment from 12 human cancer cell lines and 318 clinical samples of three urological cancers, prostate, bladder, and kidney, two genes were identified as novel DNA methylation biomarkers, PCDH17 and TCF21 (74). *PCDH17* and *TCF21* promoter methylation levels provided a sensitivity rate of 96% for prostate cancer, 92% for bladder cancer, and 67% for renal cell tumors. However, in urine samples, the sensitivity was only 38% overall with absolute specificity.

For clear cell renal carcinoma (ccRCC), by far the most common histological subtype of renal cancer, *TCF21* was an independent prognostic factor for poor survival in 186 samples of ccRCC patients, which were related to aberrant methylation of *TCF21* (75). In a ccRCC cell line, Caki-2 cells, miR-21 is upregulated compared with its expression in normal renal cells. miR-21 upregulation by pre-miR-21 decreased TCF21 expression, whereas anti-miR-21 showed the opposite effects. Moreover, siRNA-TCF21 downregulates the expression of KISS1 and the invasion ability in Cak-1 cells, suggesting that aberrantly expressed miR-21 regulates the invasion pathway through TCF21-KISS1 association in renal cell carcinoma (30). In addition, *TCF21* overexpression in ccRCC 786-O cells augmented E-cadherin expression (64).

In children, clear cell sarcoma of the kidney (CCSK) is a rare tumor with an unknown molecular pathogenesis. Analysis of 13 CCSKs showed no chromosomal copy number changes or somatic variants but identified promoter hypermethylation and low expression of *TCF21* in 12 CCSKs. TARID, the lncRNA responsible for *TCF21* demethylation, was undetectable in most CCSK samples, suggesting that *TCF21* hypermethylation and reduced TARID expression are involved in the pathogenic pathway of CCSK (76).

Similar to miR-21 activity in ccRCC, in human invasive bladder cancer cells, UMUC3 and T24T cell lines, miR-3648 inhibits TCF21 protein expression by reducing its mRNA stability. Additionally, the increase in miR-3648 regulates the TCF21/KISS1 association, resulting in the promotion of invasion and metastasis of human bladder cancer (32). In human ovarian cancer, miR-205 is increased, resulting in inhibition of *TCF21*, which is a direct target of miR-205, and promotion of cell invasion of the ovarian cell lines OVCAR-5, OVCAR-8, and SKOV-3 (31). Li et al. (77) described that *TCF21* can be a transcriptional target of p53 in response to hypoxia in uterine corpus endometrial carcinoma (UCEC) carrying wild type p53. Moreover, TCF21 interferes with the MAPK pathway by reducing ERK levels in UCEC cells expressing higher levels of *TCF21*.

## TCF21 in breast cancer

Resembling the tumors described to date, *TCF21* is less expressed in human breast cancer cell lines and tissues and is associated with larger tumor mass size and lymphoid metastases (78). Overexpression of *TCF21* in MDA-MB-231 breast cancer cells decreased cell proliferation, invasion, and migration capacity and inhibited angiogenesis, epithelial-mesenchymal transition, and apoptosis (79). The TCF21 mechanism of action in human breast tumor cell lines showed that TCF21 is able to regulate aberrant estrogen receptor-α signaling (Erα), an important factor for the progression of breast cancer, thereby reducing its functionality. The SUMOylation of TCF21 at lysine residue 24 (K24) by the small ubiquitin-like modifier SUMO1 stabilizes the TCF21 protein and enhances its interaction with histone deacetylases (HDAC1/2). The SUMOylation of TCF21 represses the activity of Erα and decreases the percentage of S-phase cells (80).

Analysis of the genetic polymorphism of *TCF21* and the risk of breast cancer in Chinese women suggests that the *TCF21* rs12190287 polymorphism can regulate TCF21 expression and may serve as a potential marker for genetic susceptibility to breast cancer (81). Interestingly, the same polymorphism is considered a prognostic factor in osteosarcoma (82).

## TCF21 in adrenocortical tumors

Adrenocortical carcinoma (ACC) is a rare disease with an incidence of 1 to 2 cases per million (83). Despite few defined markers for adrenocortical tumors (ACTs), *TCF21* is less expressed in ACC than in adenomas (ACA) and normal tissue (65,66). TCF21 binds directly in the E-box promoter sequence of steroidogenic factor 1 (SF1/NR5A1), decreasing *SF-1* transcription in both the human ACC cell line (H295R) and in ACC cell culture obtained from patient tumor fragment (ACC-T36). Increased expression of *TCF21* in H295R and ACC-T36 cells and in primary culture of rat ACC decreased *SF-1/NR5A1* and steroidogenic enzyme StAR expression (65,84). In cultures of ACA cells obtained from pediatric tumor fragments, ACA-T7 cells, *TCF21* silencing by small interfering RNA led to an increase in *SF1* mRNA expression (85). Although SF-1 regulates steroid production, it is also related to adrenocortical cell proliferation (86,87). In fact, an increase in SF-1 dosage activates adrenocortical cell proliferation and induces adrenocortical neoplasia in mice (88). However, *TCF21* overexpression showed no difference in proliferative capacity in ACC cells (65). KEGG pathway analysis has shown that *BUB1B*, among other genes, is negatively correlated with *TCF21* expression in ACC (66). More recently, analysis of the combined expression of *TCF21* and *BUB1B* in adult and pediatric adrenocortical tumors showed a negative correlation between the expression levels of *TCF21* and *BUB1B* in adult ACCs. Additionally, the combined expression of *TCF21* and *BUB1B* was a predictor of overall survival and was able to separate ACCs into two subgroups: one with poor prognosis and the other with good prognosis. In agreement with the observation that pediatric ACT appear to have a biphasic age distribution with a poor clinical outcome in the group aged >5 years, it was found that the combination of *TCF21* and *SF1* was a good predictor of malignancy for children less than 5 years old (65).

## TCF21 in other tumors

By using quantitative DNA methylation analysis in melanoma biopsies of patients and in their derived cell lines, it was demonstrated that *TCF21* expression is downregulated in metastatic melanoma by promoter hypermethylation. Moreover, *TCF21* promoter DNA methylation is correlated with decreased survival in metastatic melanoma. Functionally, TCF21 binds to the *KISS1* promoter, a melanoma metastasis-suppressor gene, increasing its expression. Finally, overexpression of *TCF21* inhibits the motility of the C8161 human melanoma cell line (18).

In colorectal and gastric cancer cells, downregulation of *TCF21* by hypermethylation induces cell proliferation, migration, and invasion, probably through inactivation of PI3K/AKT signaling (Figure 1). The overexpression of *TCF21* in two human colorectal tumor cells, HCT116 and HT29, increased KISS1 protein expression and reduced the expression of both metalloproteinases MMP2 and MMP9 (Figure 1), which are involved in cell invasion (89). As in the vast majority of tumors analyzed, *TCF21* is downregulated in esophageal squamous carcinoma (ESCC) and correlated with poor prognosis. Enhancement of *TCF21* expression levels in ESCC cells inhibits migration, invasion, and proliferation, as well as epithelial-mesenchymal transition, which may be partly through the increase in Kiss-1 and downregulation of TWIST expression (90). As the epigenetic regulation of *TCF21* through different levels of methylation is a common observation in different malignancies, these data are summarized in Table 2.

**Table 2:** Transcription factor 21 (TCF21) in tumorigenesis.

TCF21 status/function	Tumor	References
**Epigenetic modification**		
Promoter hypermethylation	lung, head and neck squamous cell carcinomas, renal cell, bladder, prostate, melanomas, colorectal, clear cell sarcoma of the kidney	26,63,68 [Bibr B69],^70^,^74^,^76^
rs12190287 polymorphism	breast, osteosarcoma	81,82
**Clinical biomarker**		
Prognostic factor	lung adenocarcinoma, clear cell renal cell carcinoma, adult ACTs, metastatic melanoma, colorectal, esophageal squamous cell carcinoma	18,66,70,75,89,90
Diagnostic factor	bladder, prostate, renal, pediatric ACTs	66,74
Tumor mass size	breast	78
Lymphoid metastases	breast, colorectal	78
**Biological effect**		
Reduction of tumor growth	gastric	67
Cell death by apoptosis	NSCLC	72
Decreased cell viability	NSCLC	72
Apoptosis reduction	breast	79
Inhibition of cell invasion	NSCLC, breast, colorectal, gastric, esophageal squamous cell carcinoma	67,68,72,79,89,90
Inhibition of cell migration	renal, breast, melanomas, colorectal, gastric, esophageal squamous cell carcinoma	18,64,67,68,79,89,90
Inhibition of cell proliferation	breast, colorectal, gastric, esophageal squamous cell carcinoma	67,68,79,89,90
Angiogenesis inhibition	breast	79

TCF21: transcription factor 21; ACTs: adrenocortical tumors; NSCLC: non-small-cell lung carcinoma cells.

## Conclusion

Taken together, these data demonstrate that TCF21 is a tumor suppressor gene epigenetically regulated by promoter methylation. TCF21 is capable of substantially interfering with important biological processes in migration, invasion, and metastasis. However, functional studies on the main pathways of TCF21 action and their partner molecules in development and tumorigenic processes are lacking. Further studies are warranted to assign to the transcription factor TCF21 the condition of tumor biomarkers and therapeutic targets.
